# A *Bacillus subtilis* xylanase improves nutrient digestibility, intestinal health and growth performance of broiler chickens undergoing an intestinal challenge

**DOI:** 10.1016/j.psj.2025.104908

**Published:** 2025-02-17

**Authors:** Catarina Stefanello, Yuri K. Dalmoro, Heitor V. Rios, Marcia S. Vieira, Mariana L. Moraes, Otoniel F. Souza, Matheus P. Araujo, Thais B. Stefanello, Raquel S. García, Christelle Boudry, Ludovic Lahaye

**Affiliations:** a Department of Animal Science, Federal University of Santa Maria, Santa Maria, RS, Brazil.; b Jefo Nutrition Inc., Saint-Hyacinthe, Quebec, Canada.; c Belfeed N.V., Andenne, Belgium.

**Keywords:** Bacterial xylanase, Energy reduction, Intestinal permeability, Short-chain fatty acid

## Abstract

The objective of this experiment was to evaluate the effects of a *Bacillus subtilis* xylanase on nutrient digestibility, intestinal health, and growth performance of broiler chickens undergoing an intestinal challenge. Nine hundred one-day-old Cobb 500 male chicks were randomly distributed into three dietary treatments with 10 replicates (30 birds/pen) until 42 d. Treatments were: Control (**CT**) diet, energy reduction (**ER**) diet, and the ER diet supplemented with *B. subtilis* endo 1,4-β-xylanase at 100 g/t (**ER+xylanase**). The ER diets had metabolizable energy reductions as per manufacturer guidelines: 80, 82, 87, and 92 kcal/kg for pre-starter, starter, grower, and finisher, respectively. All birds were challenged with *Eimeria* spp. on d 1 and *Clostridium perfringens* on d 12-14. Feed intake, body weight gain (**BWG**), and feed conversion ratio (**FCR**) were evaluated weekly. On d 17, the intestinal permeability was determined using fluorescein isothiocyanate-dextran (**FITC-d**) as marker. On d 21, I See Inside (**ISI**) methodology assessed intestinal alterations. On days 21 and 42, nutrient digestibility and cecal short-chain fatty acids (**SCFA**) concentrations were calculated. Data underwent ANOVA or Kruskal-Wallis tests, followed by Fisher or Dunn tests, respectively. From 0 to 42 d, broilers fed the ER+xylanase diet had greater (*P* = 0.046) BWG than birds fed the ER diet, while FCR showed improvements in CT and ER+xylanase diets. Broilers fed the ER+xylanase diet had improved intestinal integrity with the lowest (*P* = 0.017) serum FITC-d concentration and highest (*P* = 0.057) Mucin-2. Pancreas ISI scores were worsened (*P* = 0.034) in CT and ER diets compared to ER+xylanase diet. Ileal digestibility of DM and energy, and SCFA were greater (*P* ≤ 0.006) in broilers fed the ER+xylanase diet compared to ER diet. In conclusion, the *B. subtilis* xylanase supplementation improved nutrient digestibility, enhanced intestinal barrier integrity and increased SCFA concentrations which improved growth performance in broilers undergoing an intestinal challenge. Results indicated that this xylanase was able to compensate the energy reduction in corn-soy diets fed to broiler chickens.

## Introduction

The supplementation of xylanase has been increasingly used worldwide, and there are several commercial products available for poultry diets. Exogenous xylanases can be originated from different microorganisms such as bacteria and fungi which can greatly influence its mode of action (Moers et al., 2003). In this context, xylanase products can present variable recommended inclusion levels, units of enzyme activity, substrate affinity and resistance to xylanase inhibitors (Gebruers et al., 2008). More technology has been applied to obtain new generations of feed enzymes and different beneficial effects of xylanase supplementation can be expected when used in feed formulations for broilers (Ravindran and Son, 2011).

*Bacillus subtilis* xylanase hydrolyzes arabinoxylan (**AX**) polymers in the gastrointestinal tract and it generates products that vary according to the substrates present in ingredients. The AX present in corn and soybean meal (**SBM**) are not properly hydrolyzed by poultry due to the lack of endogenous enzymes and impairs nutrient digestibility. Insoluble AX are the main components of the non-starch polysaccharides present in corn and SBM and are known as an important antinutritional factor, which entrap nutrients and make them unavailable to be digested and/or used by the animal. As previously described by Moers et al. (2003) bacterial xylanases seem to present higher affinity to insoluble fraction of AX when compared to fungal origin xylanases. Along with that, vegetable origin ingredients contain different types and concentrations of xylanase inhibitors that can impair xylanase activity. Bacterial xylanases have been shown to be little affected by these xylanase inhibitors (Gebruers et al., 2008). Thus, the positive effects of a bacterial xylanase in corn-SBM-based diets may occur due to the increased degradation of the insoluble AX obtained with the enzyme supplementation, increasing nutrient and energy utilization and reducing mucosal stressors in the small intestine.

The released digestion products from xylanase action can stimulate different digestive and fermentative processes in broiler chickens (Vandeplas et al., 2009). The main effects of bacterial xylanases that lead to improvements on broiler intestinal health, nutrient digestibility, and growth performance have been attributed to the increased hydrolysis and solubilization of plant cell wall AX along with the reduction of their molecular weight. Bacterial xylanases can release arabinoxylan-derived oligosaccharides as arabinoxylan-oligosaccharides (**AXOS**), which are more susceptible to fermentation by the cecal microbiota and can increase the production of short-chain fatty acids (**SCFA**) (Courtin et al., 2008; Kiarie et al., 2013). Thus, the beneficial effect of bacterial xylanases on the balance of the microbiota would be attributed to two main actions: (i) reducing residual nutrients available in the hindgut for the growth of potentially pathogenic bacteria and (ii) increasing the amount of AXOS that reach the hindgut, which can then be utilized by the beneficial microbiota (Vandeplas et al., 2009).

Nowadays, more attention has been given to intestinal health, and different models of intestinal challenge have been studied. Additive supplementation in poultry diets has been increasingly used with the aim of improving intestinal health and growth performance. It was previously reported that *B. subtilis* xylanase improves the feed efficiency of broilers under an intestinal challenge with *Salmonella* Typhimurium (Vandeplas et al., 2009). These effects were attributed to the AX degradation and the reduced concentration of potentially pathogenic bacteria population due to the improved environmental gut conditions. For this reason, an additional beneficial effect of *B. subtilis* xylanase can be expected in the presence of an intestinal challenge or pathogenic bacteria (Vandeplas et al., 2009). Challenges with *Eimeria* spp. and *Clostridium perfringens* have been shown to provoke an intestinal dysbiosis in experiments. In such trials, the reduced broiler growth performance has been attributed to severe intestinal inflammation, increased intestinal permeability, and mucosal damage caused by this challenge, as well as to the toxins produced and the consequent disruption of homeostasis (Bomba et al., 2006; Lee et al., 2011; Stefanello et al., 2020; Lee and Lillehoj, 2022).

There is a lack of data evaluating *B. subtilis* xylanase supplemented in corn-SBM-based diets for broilers as well as its effects when an imposed intestinal challenge is applied. The objective of this experiment was to evaluate the effects of a *B. subtilis* endo 1,4-β-xylanase on nutrients and energy utilization, intestinal health, contents of cecal SCFA, and growth performance of broiler chickens undergoing an intestinal challenge model with *Eimeria* spp. and *C. perfringens* and fed diets with reduced energy. We tested the hypothesis that this bacterial xylanase enhances energy and nutrient utilization while supporting intestinal health, helping animals cope more effectively with intestinal challenges, which leads to improved growth performance.

## Materials and methods

All procedures used in this experiment were approved by the Ethics and Research Committee of the Federal University of Santa Maria, Santa Maria, RS, Brazil (number 1499030220).

### Broiler husbandry and intestinal challenge

A total of 900 one-day-old Cobb 500 male broiler chicks vaccinated against Marek's disease at the hatchery were randomly placed into 30 floor pens (30 birds/pen; 10 birds/m²) in a climate-controlled poultry house at the Federal University of Santa Maria experimental facilities. Each experimental pen had five nipple drinkers and one tubular feeder with 15 kg capacity. Water and mash feeds were provided *ad libitum*. New wood shavings were used as litter. The temperature was in accordance with the primary breeder's recommendation (Cobb-Vantress, 2018) to maintain bird comfort throughout the study using lamps, automatically controlled exhaust fans, and pad cooling when needed. Lighting was continuous until day 14, with a 16L:8D cycle light program used afterwards.

On day 1, all chicks were individually eye dropped with a commercial coccidial vaccine (10 × manufacturer recommended dose) containing *Eimeria acervulina, E. brunetti, E. maxima, E. mitis, E. necatrix, E. praecox*, and *E. tenella*. On days 12, 13, and 14, birds were challenged via individual oral gavage with 1 mL/bird of *C. perfringens* at 1.0 × 10^8^ cfu/mL (MercoLab, Cascavel, PR, Brazil). This challenge model was used to induce an intestinal dysbiosis to evaluate the effects of the bacterial xylanase on promoting intestinal health, nutrient digestibility, and performance in animals in suboptimal health conditions. The 10 × standard dosage of cocci vaccine was used to cause damage in broiler intestinal mucosa affecting intestinal homeostasis and immune system. These conditions create a better environment for the proliferation of *C. perfringens*. This experimental intestinal challenge was already used in many other studies with similar objectives (Belote et al., 2018; Belote et al., 2019; Bortoluzzi et al., 2019; Sanches et al., 2019; Stefanello et al., 2020).

### Experimental design, treatments and diets

Broilers were fed pre-starter (0 to 7 days), starter (7 to 21 days), grower (21 to 35 days), and finisher (35 to 42 days) corn-SBM-based experimental feeds. Birds were distributed into three treatments with 10 replicate pens in a completely randomized design. The dietary treatments were: Control (**CT**) diet; energy reduction (**ER**) diet; and ER diet supplemented with *Bacillus subtilis* xylanase at 100g/t (**ER+xylanase**). The CT diet was formulated based on ingredient and nutrient levels (broilers of regular performance) as shown in Table 1 (Rostagno et al., 2017). Celite at 1% was used as indigestible marker and it was included in starter and finisher diets 48h prior to ileal digesta collection on days 21 and 42.

**Table 1:** Ingredient and nutrient composition of the control and energy reduction (ER) experimental diets (as-fed basis).

Item	Pre-starter (0 to 7 days)		Starter (7 to 21 days)		Grower (21 to 35 days)			Finisher (35 to 42 days)		
	Control	ER^1^	Control	ER	Control	ER		Control		ER
Ingredients, %										
Corn	48.99	50.25	51.32	52.72	57.64	59.16		63.86		65.50
Soybean meal	43.82	43.62	41.44	41.21	35.22	34.98		29.89		29.63
Soybean oil	3.52	2.46	3.93	2.75	4.27	2.99		3.85		2.47
Dicalcium phosphate	1.04	1.04	0.83	0.83	0.57	0.57		0.28		0.28
Limestone	1.38	1.38	1.27	1.27	1.14	1.14		1.00		1.00
Salt	0.52	0.52	0.52	0.52	0.50	0.50		0.47		0.47
DL-Met, 99%	0.34	0.34	0.32	0.32	0.27	0.27		0.23		0.23
L-Lys HCl, 78%	0.13	0.13	0.13	0.14	0.15	0.15		0.17		0.17
L-Thr, 98.5%	0.05	0.05	0.05	0.05	0.04	0.04		0.03		0.03
Choline chloride, 60%	0.03	0.03	0.04	0.04	0.05	0.05		0.07		0.07
Vit. and Min. premix^2^	0.18	0.18	0.15	0.15	0.15	0.15		0.15		0.15
Phytase^3^	0.005	0.005	0.005	0.005	0.005	0.005		0.005		0.005
Calculated nutritional composition, % or as shown										
ME, kcal/kg	2,975	2,895	3,050	2,968	3,150		3,063		3,200	3,108
Crude protein^4^	24.10	24.10	23.20	23.20	20.86		20.86		18.92	18.92
Ca	0.97	0.97	0.88	0.88	0.76		0.76		0.63	0.63
Available P	0.46	0.46	0.42	0.42	0.36		0.36		0.30	0.30
Na	0.22	0.22	0.22	0.22	0.21		0.21		0.20	0.20
K	0.96	0.96	0.92	0.92	0.83		0.83		0.75	0.75
Cl	0.40	0.40	0.41	0.41	0.40		0.40		0.40	0.40
Choline, mg/kg	1,600	1,600	1,600	1,600	1,500		1,500		1,500	1,500
Digestible Lys	1.307	1.307	1.256	1.256	1.124		1.124		1.014	1.014
Digestible Met + Cys	0.97	0.97	0.93	0.93	0.83		0.83		0.75	0.75
Digestible Thr	0.86	0.86	0.83	0.83	0.74		0.74		0.67	0.67
Digestible Trp	0.28	0.28	0.27	0.27	0.24		0.24		0.21	0.21
Digestible Arg	1.53	1.53	1.46	1.46	1.29		1.29		1.15	1.15
Digestible Val	1.01	1.01	0.97	0.97	0.87		0.87		0.78	0.78
Digestible Ile	0.95	0.95	0.91	0.91	0.80		0.80		0.72	0.72
Digestible Leu	1.82	1.82	1.76	1.76	1.63		1.63		1.52	1.52

1 Energy reduction diet = diet formulated with reductions in metabolizable energy at 80 kcal/kg (from 0 to 7 days), 82 kcal/kg (from 7 to 21 days), 87 kcal/kg (from 21 to 35 days), and 92 kcal/kg (from 35 to 42 days).

2 Composition per kilogram of feed: vitamin A, 8,000 UI; vitamin D3, 2,000 UI; vitamin E, 30 UI; vitamin K3, 2 mg; thiamine, 2 mg; riboflavin, 6 mg; pyridoxine, 2.5 mg; cyanocobalamin, 0.012 mg, pantothenic acid, 15 mg; niacin, 35 mg; folic acid, 1 mg; biotin, 0.08 mg; iron, 40 mg; zinc, 80 mg; manganese, 80 mg; copper, 10 mg; iodine, 0.7 mg; selenium, 0.3 mg.

3 Phytase with 20,000 fungal phytase units/g, using available P and total Ca (g/kg) matrix values of 29,900 and 34,445, respectively (Ronozyme HiPhos, Novozymes, Denmark).

4 Analyzed crude protein was 23.9%, 23.1%, 20.6%, and 18.9% in pre-starter, starter, grower, and finisher diets, respectively.

The ER diets were formulated with metabolizable energy reductions according to the manufacturer recommendation, being: 80, 82, 87, and 92 kcal/kg for pre-starter, starter, grower, and finisher, respectively. The ER+xylanase diet was supplemented with 100 g/t xylanase (Belfeed B1100 MP, Belfeed N.V., Andenne, Belgium) originated from *B. subtilis* (LMG S-15136). This is an endo 1,4-β-xylanase (105 IU/g) with optimal activity at pH 6 to 7. One international unit is the amount of enzyme that liberates 1 μmol of xylose from birchwood xylan per minute at pH 4.5 and 30°C (Vandeplas et al., 2009).

### Growth performance and footpad dermatitis

On the initial day (day 0), chicks were weighed in groups of 30 birds per pen, with an average weight of 46 ± 1 g. Body weight and feed intake (**FI**) per pen were recorded at 7, 14, 21, 28, 35, and 42 days of age. Mortality data were collected daily. Body weight gain (**BWG**) and feed conversion ratio adjusted for the weight of deceased birds (**FCR**) were calculated for each replicate group, as well as by week and feeding phase.

On day 42, five broilers from each pen, individually weighed and selected to match the average BW of the pen, were examined for signs of footpad dermatitis on both feet. An average lesion score for both feet was calculated for each bird. A four-point lesion scale was used, with scores defined as follows: 0 (no visible lesions), 1 and 2 (minimal evidence), and 3 and 4 (evidence) (WelfareQuality, 2009).

### Intestinal permeability measurement

On day 17, one bird per pen, selected to match the pen's average BW and individually weighed, received 4.16 mg/kg BW of fluorescein isothiocyanate-dextran (**FITC-d**; 3–5 kDa; 4,000 molecular weight; Sigma-Aldrich, Saint Louis, MO, USA), which was dissolved in 1 mL of Milli-Q water via oral gavage. After a withdrawal period of 1.5 h, blood samples were collected from the brachial wing vein and centrifuged to isolate serum. Fluorescence levels in the diluted serum samples (1:1 with PBS) were measured, and FITC-d concentration (µg/mL) was calculated based on a standard curve using blood from non-gavaged birds (Gilani et al., 2018) as described in previous studies from the research group (Stefanello et al., 2020; Stefanello et al., 2022). FITC-d, a high-molecular-weight molecule, serves as a marker of intestinal barrier integrity. Increased levels of FITC-d in blood samples indicate greater intestinal mucosa permeability.

### Gene expression of intestinal health biomarkers

On day 21, jejunal mucosa samples were collected by scraping from two birds per pen, with samples pooled per pen to assess mRNA expression levels of specific genes. These included interleukins IL-10 and IL-12 as markers of intestinal inflammation, mucin-2 (**MUC-2**) as an indicator of intestinal barrier integrity, claudin-1 (**CLDN1)**, and claudin-5 (**CLDN5**) as markers of tight junction permeability regulation. Gene expression was quantified via real-time quantitative PCR (**qRT-PCR**), following the protocol outlined by Stefanello et al. (2020). Total RNA was isolated and purified from a homogenized sample of approximately 100 mg of intestinal mucosa using Tri Reagent (Trizol; Sigma-Aldrich, St. Louis, MO, USA). The RNA extracts were treated with DNaseI to remove any DNA contaminants, quantified, and assessed for purity through spectrophotometry. After RNA extraction, complementary DNA (**cDNA**) Synthesis was performed using 1 μg of RNA per reaction. The qPCR reaction was performed by diluting the genetic material in sterile MilliQ water, and the targets quantified using Sybr Green PCR Master Mix in a qPCR thermocycler. The cycling conditions used were 95°C for 10 min, followed by 40 cycles of 95°C for 15s and 60°C for 1 min. Two normalization genes were used as internal controls. The results were normalized by ΔΔCt (ΔCt/mean ΔCt Control).

### Morphometry and digestive tract analyses

On day 21, the intestinal health of two animals per replicate was evaluated for macroscopic and/or histologic alterations in the pancreas, duodenum, jejunum, ileum, and cecum, utilizing the I See Inside (**ISI**) methodology (Kraieski et al., 2017; Belote et al., 2018). Following the ISI methodology, scores were given for each observed condition in each organ, ranging from 0 to 3. Score 0 corresponds to absence of lesions, score 1 refers to alteration up to 25% of the area, score 2 presents alteration ranging from 25 to 50% of the area, and score 3 is reported when alteration extent is more than 50% of the area. The score given to each alteration is then multiplied by an impact factor that ranges from 1 to 3 to obtain the ISI score. The impact factor is determined by the methodology based on the reduction of functional capacity of the organ, with 3 representing the most impactful alteration and 1 representing the least. The ISI score of each organ is summed to obtain the total ISI score. More detailed information on the ISI methodology, measurements of lesion intensity, impact factor, and scores can be found in the publications of Belote et al. (2018), Sanches et al. (2019), and Stefanello et al. (2020).

The macroscopic analysis was evaluated in two birds per pen (individually weighed and within the average BW of the pen) by the same trained observer and given scores for pancreas alterations (hypotrophy and hypertrophy), and for alterations in duodenum, jejunum, ileum and cecum due to *Eimeria* lesions, necrosis (jejunum only), inflammation process at serosa or mucosa, decreased muscular tonus (jejunum only), gas presence (cecum only) and cell debris and thick mucus at mucosa.

For the histological analysis, samples from the proximal ileum (from the same two birds per pen used in the macroscopic analysis) were collected and preserved in Davidson's solution. The samples underwent standard histological processing, including dehydration, infiltration, and embedding in paraffin. Sections were then cut at 5 μm thickness and stained with hematoxylin and eosin, as well as Alcian Blue. In each bird, approximately 20 intestinal villi were analyzed per slide using a light microscope (Nikon Eclipse E200, Sao Paulo, SP, Brazil). The assessed ISI variables included lamina propria and epithelial thickness, inflammatory cell infiltration in the lamina propria, proliferation of enterocytes and Goblet cells, infiltration of plasma cells in the epithelium, and presence of oocysts. The scores for each variable were summed to calculate the overall ISI score.

### Cecal short-chain fatty acids measurement

On days 21 and 42, four birds per pen (with the average BW of the pen) were euthanized for cecal content collection to determine the concentration of SCFAs following the methodology presented by Leal et al. (2021). Distilled water (1 mL) was added to 1 g of cecal content, homogenized for 30 s, and centrifuged at 11,300 × g for 5 min. A total of 200 μL of the supernatant was added to 200 μL formic acid and homogenized. Following, 100 μL of this solution was diluted in 100 μL internal standard of 3-octanol and 1 µL from this extract was injected in a gas chromatograph equipment. The column used was CP-WAX 52 CB and the temperature was adjusted to 80°C for 1 min, increasing to 120°C at 8°C/min, and increasing at 15°C / min until 230°C. Limit of detection (**LOD**), limit of quantification (**LOQ**), selectivity, linearity, linear range, repeatability, and accuracy were used to validate the method for the following compounds: acetic, propionic, butyric, isovaleric, valeric, and isobutyric acids. Results of each SCFA were expressed in mmol/kg of cecal content.

### Ileal digestibility analysis

Ileal contents from the distal two-thirds of the ileum were flushed with distilled water into containers, collected per experimental unit, and stored at −20°C. These samples were obtained from the same birds from which cecal contents were collected on days 21 and 42. Before analysis, digesta samples were dried in a forced-air oven at 55°C and then ground. Feed and ileal digesta samples were further dried at 105°C for 16 h (AOAC, 2006) to assess DM content. Nitrogen was measured using the combustion method, while gross energy was determined with a bomb calorimeter. Acid-insoluble ash was extracted from both feed and ileal digesta to determine the indigestible marker. Apparent ileal digestibility of DM, protein, and energy as well as ileal digestible energy (**IDE**, in kcal/kg), on DM basis, were calculated as previously described (Kong and Adeola, 2014; Stefanello et al., 2020).

### Statistical analysis

Data was subjected to ANOVA using the MIXED procedure of SAS Institute version 9.4 (SAS, 2015) when parametric and mean separation was done with the Fisher test. Non-parametric data was submitted to Kruskal-Wallis followed by post-hoc Dunn test. Significant differences among treatment means were declared when *P* ≤ 0.05, while tendencies were declared when 0.05 < *P* ≤ 0.10.

## Results

From 0 to 21, 7 to 21 and 14 to 21 days, broilers fed CT or ER+xylanase diets had greater BWG than broilers fed the ER diet (*P* ≤ 0.05; Table 2). The FCR tended to improve in broilers fed the ER+xylanase diet compared to broilers fed the ER diet from 14 to 21 days (*P* = 0.068). From 21 to 28 days, broilers fed the CT diet tended to present lower FCR than those fed the ER diet (*P* = 0.069), not differing from ER+xylanase diet. In the overall period, broilers fed ER+xylanase diet presented greater BWG than those fed the ER diet (*P* ≤ 0.05), being similar to those from CT diet. A trend for better FCR was observed in broilers fed ER+xylanase diet and CT diet when compared to the ER diet (*P* = 0.100). Dietary treatments did not significantly affect mortality throughout the study nor footpad dermatitis at 42 days.

**Table 2:** Growth performance and footpad dermatitis of broiler chickens undergoing an intestinal challenge and fed diets not supplemented or supplemented with a *Bacillus subtilis* xylanase^1^.

Item^2^	Control	ER^3^	ER+xylanase	SEM	*P*-value
0 to 7 days					
FI, g	155	158	156	0.9	0.371
BWG, g	132	129	132	1.4	0.606
FCR	1.180	1.240	1.187	0.015	0.204
7 to 14 days					
FI, g	440	442	459	5.0	0.236
BWG, g	341	331	340	2.4	0.133
FCR	1.294	1.338	1.348	0.015	0.330
14 to 21 days					
FI, g	758	760	759	7.4	0.995
BWG, g	550^a^	528^b^	554^a^	4.4	0.031
FCR	1.381^xy^	1.441^x^	1.369^y^	0.014	0.068
21 to 28 days					
FI, g	1,019	988	986	8.3	0.203
BWG, g	677	649	649	8.0	0.274
FCR	1.481^y^	1.525^x^	1.520^xy^	0.009	0.069
28 to 35 days					
FI, g	1,343	1,395	1,384	18.2	0.481
BWG, g	758^b^	760^b^	823^a^	11.7	0.028
FCR	1.779	1.839	1.688	0.032	0.142
35 to 42 days					
FI, g	1,342	1,337	1,368	24.5	0.867
BWG, g	632	604	651	15.3	0.468
FCR	2.139	2.222	2.117	0.039	0.519
0 to 21 days					
FI, g	1,354	1,360	1,374	11.4	0.777
BWG, g	1,022^a^	987^b^	1026^a^	6.0	0.009
FCR	1.325	1.379	1.338	0.012	0.160
7 to 21 days					
FI, g	1,198	1,202	1,218	11.2	0.768
BWG, g	890^a^	859^b^	895^a^	5.8	0.017
FCR	1.347	1.401	1.361	0.012	0.191
21 to 35 days					
FI, g	2,362	2,383	2,370	21.5	0.921
BWG, g	1,451	1,409	1,473	13.9	0.164
FCR	1.630	1.692	1.614	0.017	0.152
21 to 42 days					
FI, g	3,704	3,720	3,738	34.3	0.924
BWG, g	2,083	2,013	2,124	23.3	0.146
FCR	1.782	1.850	1.766	0.020	0.201
0 to 42 days					
FI, g	5,057	5,081	5,112	38.6	0.854
BWG, g	3,105^ab^	3,000^b^	3,150^a^	25.8	0.046
FCR	1.631^y^	1.694^x^	1.625^y^	0.015	0.100
Mortality, %	2.0	3.0	3.0	0.4	0.642
Footpad dermatitis^4^					
Scores at 42 days	3.22	3.36	3.10	0.05	0.229

a,b Means with different superscripts differ *P* ≤ 0.05 and were considered significantly different based on Fisher LSD test.

x,y Means with different superscripts differ 0.0501 ≤ *P* ≤ 0.10 and were considered tendencies.

1 Control: formulated to meet nutritional requirements of broilers (Rostagno et al., 2017). ER: energy reduction diet. ER+xylanase: energy reduction diet + 100 g/t of a *Bacillus subtilis* xylanase. Xylanase = *Bacillus subtilis* supplemented at 100 g/t (Belfeed B1100MP, Belfeed N.V., Andenne, Belgium).

2 FI = feed intake, BWG = body weight gain, FCR = feed conversion ratio.

3 Metabolizable energy reductions were: 80 kcal/kg (0 to 7 days), 82 kcal/kg (7 to 21 days), 87 kcal/kg (21 to 35 days), and 92 kcal/kg (35 to 42 days).

4 Scores of footpad dermatitis were obtained from 5 birds per pen with 10 replicate pens each treatment.

Broilers fed ER+xylanase diet presented the highest DM and energy digestibility at 21 and 42 days (*P* ≤ 0.05; Table 3). The IDE was higher for ER+xylanase when compared to ER at 21 and 42 days (*P* ≤ 0.05), which was not different from CT diet. Additionally, there was a tendency for higher protein digestibility in 42-day-old broilers fed the ER+xylanase diet when compared to those fed the ER diet (*P* = 0.096).

**Table 3:** Nutrient digestibility and ileal digestible energy of broiler chickens undergoing an intestinal challenge and fed diets not supplemented or supplemented with a *Bacillus subtilis* xylanase^1^.

Item	Control	ER^2^	ER+xylanase	SEM	*P*-value
Apparent ileal digestibility, 21 days					
Dry matter, %	71.5^b^	69.6^b^	75.3^a^	0.68	0.001
Crude protein, %	84.7	82.7	84.4	0.45	0.149
Energy	69.2^b^	67.2^b^	73.6^a^	0.81	0.002
IDE^3^, kcal/kg	2,858^ab^	2,745^b^	2,994^a^	33.5	0.006
Apparent ileal digestibility, 42 days					
Dry matter, %	75.0^b^	73.7^b^	77.2^a^	0.46	0.003
Crude protein, %	83.1^xy^	81.3^y^	83.6^x^	0.45	0.096
Energy	73.6^b^	71.0^c^	76.0^a^	0.50	0.001
IDE, kcal/kg	3,051^ab^	2,960^b^	3,075^a^	20.5	0.049

a,b,c Means with different superscripts differ *P* ≤ 0.05 and were considered significantly different based on Fisher LSD test.

x,y Means with different superscripts differ 0.0501 ≤ *P* ≤ 0.10 and were considered tendencies.

1 Control: formulated to meet nutritional requirements of broilers (Rostagno et al., 2017). ER: energy reduction diet. ER+xylanase: energy reduction diet + 100 g/t of a *Bacillus subtilis* xylanase. Xylanase = *Bacillus subtilis* supplemented at 100 g/t (Belfeed B1100MP, Belfeed N.V., Andenne, Belgium).

2 Energy reductions were: 80 kcal/kg (from 0 to 7 days), 82 kcal/kg (from 7 to 21 days), 87 kcal/kg (from 21 to 35 days), and 92 kcal/kg (from 35 to 42 days).

3 IDE = ileal digestible energy on dry matter basis. Means of ileal digestibility were obtained from 10 replicate pens of 4 birds per replicate pen in each age.

At 21 days, broilers fed the CT diet or the ER+xylanase diet had greater cecal concentration of acetic, propionic, and butyric acids when compared to broilers fed the ER diet (*P* ≤ 0.05; Table 4). At 42 days, propionic acid from broilers fed the ER+xylanase diet was similar to those from the CT diet, with broilers receiving the CT diet tending to present greater propionic acid compared to those fed the ER diet (*P* = 0.067). No differences among treatments were observed for isobutyric, isovaleric, and valeric cecal acids concentration on days 21 and 42 and acetic and butyric acids on day 42.

**Table 4:** Cecal short-chain fatty acids content (mmol/kg) of broiler chickens undergoing an intestinal challenge and fed diets not supplemented or supplemented with a *Bacillus subtilis* xylanase^1^.

Item	Control	ER^2^	ER+xylanase	SEM	*P*-value
Short-chain fatty acids, 21 days					
Acetic	80.86^a^	71.21^b^	80.27^a^	1.313	0.001
Propionic	10.02^a^	7.69^b^	11.04^a^	0.382	0.001
Butyric	20.89^a^	14.90^b^	19.84^a^	0.781	0.001
Isobutyric	1.13	1.08	1.30	0.064	0.329
Valeric	1.72	1.59	1.70	0.119	0.891
Isovaleric	1.21	1.07	1.16	0.079	0.754
Short-chain fatty acids, 42 days					
Acetic	68.25	63.74	67.48	1.966	0.622
Propionic	19.36^x^	15.96^y^	17.87^xy^	0.607	0.067
Butyric	15.95	13.93	15.93	0.594	0.289
Isobutyric	1.33	1.31	1.31	0.033	0.969
Valeric	2.02	1.84	2.03	0.080	0.566
Isovaleric	1.58	1.60	1.78	0.055	0.292

a,b Means with different superscripts differ *P* ≤ 0.05 and were considered significantly different based on Fisher LSD test.

x,y Means with different superscripts differ 0.0501 ≤ *P* ≤ 0.10 and were considered tendencies.

1 Control: formulated to meet nutritional requirements of broilers (Rostagno et al., 2017). ER: energy reduction diet. ER+xylanase: energy reduction diet + 100 g/t of a *Bacillus subtilis* xylanase. Xylanase = *Bacillus subtilis* supplemented at 100 g/t (Belfeed B1100MP, Belfeed N.V., Andenne, Belgium).

2 Energy reductions were: 80 kcal/kg (from 0 to 7 days), 82 kcal/kg (from 7 to 21 days), 87 kcal/kg (from 21 to 35 days), and 92 kcal/kg (from 35 to 42 days).

Broilers fed the ER+xylanase diet had improved intestinal integrity with the lowest serum FITC-d concentration (*P* ≤ 0.05; Table 5) and tended to present the highest MUC-2 gene expression (*P* = 0.057). No statistical differences were observed for expressions of IL-10, IL-12, CLDN1, and CLDN5 at 21 days of age. The ISI macroscopic evaluation showed a tendency for a lowest score (lower alterations) on pancreas hypotrophy for broilers fed ER+xylanase (*P* = 0.083; Table 6). In addition to that, a lower total ISI score for pancreas was observed in broilers fed to the ER+xylanase diet compared to birds fed to the ER diet (*P* ≤ 0.05), not differing from CT diet. The ER+xylanase diet presented similar scores for inflammation process at duodenum mucosa and serosa compared to the CT diet, which presented lower alterations than ER (*P* ≤ 0.05). Broilers fed energy reduced diets regardless of the supplementation of *B. subtilis* xylanase presented lower cell debris and thick mucus at ileal mucosa when compared to CT diet (*P* ≤ 0.05). Dietary treatments did not affect macroscopic overall ISI scores for duodenum, jejunum, ileum, and cecum and had no effect in the macroscopic ISI total score. The histological ISI of ileum was not affected by the dietary treatments (Table 7).

**Table 5:** Intestinal integrity and gene expression of cytokines, tight junction proteins, and MUC-2 of broiler chickens undergoing an intestinal challenge and fed diets not supplemented or supplemented with a *Bacillus subtilis* xylanase^1^.

Item	Control	ER^3^	ER+xylanase	SEM	*P*-value ANOVA	*P*-value Kruskall-Wallis
Intestinal integrity, 17 days						
FITC-d, µg/mL^2^	0.267^a^	0.266^a^	0.209^b^	0.010	0.017	-
Jejunal gene expression, 21 days						
IL-10	1.080	1.101	0.958	0.064	0.632	-
IL-12	1.127	1.037	0.916	0.089	-	0.552
Claudin-1	0.931	1.113	1.330	0.078	-	0.231
Claudin-5	1.106	1.047	1.423	0.094	0.198	-
Mucin-2	1.071^y^	1.099^y^	1.641^x^	0.110	0.057	-

a,b Means with different superscripts differ *P* ≤ 0.05 and were considered significantly different based on Fisher LSD or Dunn test.

x,y Means with different superscripts differ 0.0501 ≤ *P* ≤ 0.10 and were considered tendencies based on Fisher or Dunn test.

1 Control: formulated to meet nutritional requirements of broilers (Rostagno et al., 2017). ER: energy reduction diet. ER+xylanase: energy reduction diet + 100 g/t of a *Bacillus subtilis* xylanase. Xylanase = *Bacillus subtilis* supplemented at 100 g/t (Belfeed B1100MP, Belfeed N.V., Andenne, Belgium).

2 Blood samples were taken from 1 bird per pen. FITC-d = systemic fluorescein isothiocyanate-dextran.

3 Energy reductions were: 80 kcal/kg (from 0 to 7 days), 82 kcal/kg (from 7 to 21 days), 87 kcal/kg (from 21 to 35 days), and 92 kcal/kg (from 35 to 42 days).

**Table 6:** I See inside (ISI) macroscopic responses at 21 days of broiler chickens undergoing an intestinal challenge and fed diets not supplemented or supplemented with a *Bacillus subtilis* xylanase^1^.

Item^2^	Control	ER^3^	ER+xylanase	SEM	*P*-value
Pancreas					
Hypertrophic	0.00	0.20	0.00	0.05	0.131
Hypotrophic	2.10^x^	2.50^x^	1.10^y^	0.25	0.083
ISI score	2.10^ab^	2.70^a^	1.10^b^	0.25	0.034
Duodenum					
Inflammation process at serosa or mucosa	1.40^a^	0.85^b^	1.00^ab^	0.08	0.017
Cell debris and thick mucus at mucosa	2.00	2.00	1.80	0.16	0.695
*Eimeria* lesions	0.40	0.90	0.30	0.16	0.548
ISI score	3.80	3.75	3.10	0.28	0.439
Jejunum					
Necrosis	0.00	0.00	0.15	0.05	0.368
Inflammation process at serosa or mucosa	0.90	0.85	0.85	0.07	0.940
Cell debris and thick mucus at mucosa	2.90	3.10	3.30	0.19	0.832
Decreased muscular tonus	2.40	2.10	2.00	0.22	0.787
*Eimeria* lesions	0.60	0.15	0.00	0.13	0.154
ISI score	6.80	6.20	6.30	0.43	0.889
Ileum					
Inflammation process at serosa or mucosa	0.70	0.90	1.20	0.10	0.114
Cell debris and thick mucus at mucosa	0.50^a^	0.00^b^	0.00^b^	0.09	0.015
ISI score	1.20	0.90	1.20	0.12	0.441
Cecum					
Gas presence	1.10	0.95	1.20	0.08	0.601
Inflammation process at serosa or mucosa	0.50	0.00	0.00	0.10	0.146
*Eimeria* lesions	0.10	0.11	0.00	0.05	0.361
ISI score	1.70	1.05	1.20	0.14	0.443
Macroscopic ISI total score	15.60	14.60	12.90	0.64	0.188

a,b Means with different superscripts differ *P* ≤ 0.05 and were considered significantly different based on Dunn test.

x,y Means with different superscripts differ 0.0501 ≤ *P* ≤ 0.10 and were considered tendencies.

1 Control: formulated to meet nutritional requirements of broilers (Rostagno et al., 2017). ER: energy reduction diet. ER+xylanase: energy reduction diet + 100 g/t of a *Bacillus subtilis* xylanase. Xylanase = *Bacillus subtilis* supplemented at 100 g/t (Belfeed B1100MP, Belfeed N.V., Andenne, Belgium).

2 Macroscopic evaluation obtained from 2 birds per pen with 10 replicate pens each treatment.

3 Energy reductions were: 80 kcal/kg (from 0 to 7 days), 82 kcal/kg (from 7 to 21 days), 87 kcal/kg (from 21 to 35 days), and 92 kcal/kg (from 35 to 42 days).

**Table 7:** I See inside (ISI) histological responses at 21 days of broiler chickens undergoing an intestinal challenge and fed diets not supplemented or supplemented with a *Bacillus subtilis* xylanase^1^

Item^2^	Control	ER^3^	ER+xylanase	SEM	*P*-value ANOVA	*P*-value Kruskall-Wallis
Microscopic ileum						
Lamina propria thickness	2.37	2.38	2.32	0.05	0.808	
Epithelia thickness	1.20	1.17	1.19	0.02	0.696	
Enterocytes proliferation	1.10	1.05	1.07	0.01		0.419
Epithelial plasma cell infiltration	1.21	1.19	1.21	0.02	0.861	
Inflammatory infiltration in lamina propria	2.31	2.35	2.30	0.05	0.910	
Goblet cells proliferation	1.88	1.88	1.85	0.02		0.664
Congestion	0.22	0.26	0.18	0.02		0.238
Presence of oocysts	0.04	0.00	0.01	0.01		0.349
Microscopic ISI total score	10.33	10.28	10.13	0.10	0.671	

1 Control: formulated to meet nutritional requirements of broilers (Rostagno et al., 2017). ER: energy reduction diet. ER+xylanase: energy reduction diet + 100 g/t of a *Bacillus subtilis* xylanase. Xylanase = *Bacillus subtilis* supplemented at 100 g/t (Belfeed B1100MP, Belfeed N.V., Andenne, Belgium).

2 Microscopic evaluation obtained from 2 birds per with 10 replicate pens each treatment.

3 Energy reductions were: 80 kcal/kg (from 0 to 7 days), 82 kcal/kg (from 7 to 21 days), 87 kcal/kg (from 21 to 35 days), and 92 kcal/kg (from 35 to 42 days).

## Discussion

The objective of administering the coccidiosis vaccine and orally gavaging broiler chickens with *C. perfringens* was to induce an intestinal dysbiosis with a known disturbance to the intestinal homeostasis. Thus, in the present study, the experimental intestinal challenge did not result in high mortality (< 3%) nor severe clinical signs of necrotic enteritis. All broilers were challenged in the experiment and the comparison between unchallenged *vs.* challenged groups of broilers submitted to the same intestinal challenge protocol was already addressed by the research group in a previous study (Stefanello et al., 2020). That study demonstrated that the intestinal challenge was able to negatively impact intestinal health, nutrient digestibility, and broiler performance.

There is a lack of data evaluating *B. subtilis* xylanase supplemented in corn-soy based diets for broilers as well as its effects when experimental intestinal challenges are applied. The few studies found in the literature reported the effects of xylanase on wheat-soy diets (Vandeplas et al., 2009; Bautil et al., 2021) or xylanase-inhibiting proteins in sorghum and wheat (Gebruers et al., 2008). In the present study, improved BWG from 0 to 21 days was observed in broilers fed the ER+xylanase diet when compared to those fed the ER diet. Similarly, Bautil et al. (2021) evaluated the impact of xylanase type and dose on broiler performance at 11, 22, and 36 days of age. The authors observed improvements on BWG when a *B. subtilis* xylanase was supplemented in wheat-soy diets for broilers from 1 to 22 days when compared to a *Nonomuraea flexuosa* xylanase. The authors also discussed the effect of age in the observed results, indicating that an immature cecal microbiota had lower ability to ferment the dietary AX; however, the beneficial microbiota was improved when xylanase was used probably due to an increased supply of arabinoxylan-oligomers. Still, the AX can also affect the kinetics of cecal microbiota, and these findings indicated a large involvement of the age-related microbial development, which might increase BWG at later ages.

In the current experiment, BWG and FCR of broilers fed the ER+xylanase diet were improved by 5% and 4%, respectively, compared to those fed the ER diet from 0 to 42 days. Similarly, Vandeplas et al. (2009) tested a *B. subtilis* xylanase supplemented in wheat-soy diets and evaluated the enzyme's ability in mitigating the impact of a *Salmonella* Typhimurium challenge on broiler performance. They observed that broilers fed diets with xylanase supplementation had a 2% improvement in FCR from 7 to 42 days compared to those fed diets without xylanase.

Insoluble AXs are the most abundant fraction of AX in the plant cell wall of the main cereals utilized in broiler diets representing approximately 98%, 95%, and 78% of total AX present in corn, sorghum, and wheat, respectively (Choct, 1997). The insoluble AX can entrap nutrients in the polysaccharides structure, decreasing the access of endogenous digestive enzymes to nutrients. By disrupting AX polymers, xylanases may consequently increase the efficiency of nutrient digestion (Bautil et al., 2021). However, almost all the experiments were conducted with non-challenged birds and the effects of xylanase in the presence of an intestinal challenge should be further explored to better understand its benefits.

Supplementing the *B. subtilis* xylanase in energy reduced diets in the present experiment increased diet IDE by 9.1% and 3.9% at 21 and 42 days, respectively, being similar to the CT diet. The DM digestibility also improved by 8.2% and 4.8% at 21 and 42 days, respectively, when xylanase was supplemented. In addition, at 42 days, the protein digestibility tended to be 2.8% higher with xylanase supplementation when compared to broilers fed the ER diet. The improvements in nutrient digestibility agreed with the findings of Vandeplas et al. (2009) who observed better crude fiber and a tendency for higher protein digestibility in broilers facing an intestinal challenge and fed diets supplemented with *B. subtilis* xylanase when compared to broilers fed non-supplemented diets.

In the present study, xylanase improved ileal digestibility of DM and energy utilization. Bautil et al. (2021) observed that broilers fed *B. subtilis* xylanase presented a better digestibility of total AX when compared to broilers fed a diet supplemented with *N. flexuosa* xylanase, suggesting that the observed improvements were probably due to a better ileal AX digestion with a positive contribution to the fermentation process by beneficial bacteria in the ceca. These results can suggest that a beneficial microbiota and a balance in the intestinal microbial development can contribute to improve intestinal health, enhancing energy utilization and, consequently, growth performance.

There were increased concentrations of cecal acetic, propionic, and butyric acids at 21 days in the broilers fed the supplemented diet with *B. subtilis* xylanase. The SCFAs are groups of fatty acids with less than six carbons that are also produced in the hindgut by bacterial fermentation of dietary fibers, such as AXOS. Acetic, propionic, and butyric acids represent more than 95% of the total cecal SCFAs and play a significant role in the energy metabolism as well as in the regulation of intestinal health in poultry (Liu et al., 2021). The SCFAs have been associated with the inhibition of pathogen invasion and colonization, presenting antimicrobial effects, enhancing immune functions, and preventing inflammation (Ali et al., 2022). Butyric acid can be absorbed from the intestine and is an important energy source for enterocytes, being related to a healthier intestinal mucosa and improvements in the tight junction barrier. In general, SCFAs can regulate mucin production in the gut and promote the secretion of antimicrobial peptides that indirectly regulate the microbiota barrier (Liu et al., 2021). The bacterial xylanase has been demonstrated to beneficially enhance intestinal health probably through the improvement in the AXOS production and consequently the SCFA contents. Thus, feed additives that can regulate the production of SCFAs, as bacterial xylanases, are desirable to improve poultry intestinal health and feed efficiency (Ghosh et al., 2021).

In the current study, the negative effects on intestinal permeability caused by the intestinal challenge could be alleviated with the bacterial xylanase supplementation. Also, broilers fed the ER+xylanase diet had the lowest serum FITC-d, which is related to better intestinal integrity as FITC-d has a high molecular weight and reaches the broiler bloodstream when there is damage in the intestinal barrier. Broilers fed the ER+xylanase diet tended to present the highest MUC-2 gene expression. Damages in the intestinal mucosa and in tight junction proteins can lead to a reduced MUC-2 production and can result in higher intestinal permeability with increased FITC-d concentration detected in the blood. As reported by Stefanello et al. (2020), the *Eimeria* spp. and *C. perfringens* challenge, same challenged model used in the present study, increased intestinal permeability in 17-day-old broilers, decreasing nutrient digestibility and broiler performance.

In the macroscopic ISI analysis, higher scores, illustrative of higher alterations, for pancreas were observed for the ER diet when compared to the ER+xylanase diet. Alterations in pancreas including hypotrophy and hypertrophy, may be related to impairments in digestibility of nutrients caused by the presence of AX in diets along with the intestinal challenge caused by *Eimeria* spp. and *C. perfringens*. In a literature review, Jacob and Pescatore (2012) reported alterations in the size of pancreas when broiler chickens were fed non-starch polysaccharides (**NSPs**) rich diets and emphasized the effects of NSPs in modifying the secretion of pancreatic digestive enzymes affecting pancreas functionality. Considering this, the action of the xylanase on the NSPs can explain the reduction of pancreas size alterations. In addition, intestinal dysbiosis, including those provoked by pathogenic microorganisms, such as *Eimeria* spp. and *C. perfringens,* are known to affect broiler antioxidative mechanisms and increase oxidative stress (Lauridsen, 2019). Although not measured in the present study, oxidative stress can negatively affect pancreas functionality and has been indicated as a possible cause of pancreas damages during intestinal disorders (Hoerr, 1998). The reduction of the pancreas alterations in presence of the xylanase could thus also be linked to a reduction in the development of the pathogenic microorganisms. In the absence of the microbiota evaluation in this study, this should be confirmed in future research.

Both soluble and insoluble AXs forms impair nutrient digestibility and can potentially increase the amount of undigested nutrients that reach the hindgut and are used by bacterial population. Thus, the beneficial effect on nutrient digestibility and intestinal integrity observed with the use of this bacterial xylanase may be related to the hydrolyzation of soluble and insoluble AXs as discussed by Bautil et al. (2021). In addition to that, the effect of the *B. subtilis* xylanase on the AX can potentially benefit broiler microbiota probably due to the production of better fermented AXOS that reach the hindgut and to a positive modulation of gut microbiota as observed by Vandeplas et al. (2009).

Previously publications reported a positive effect of xylanase on broiler performance, also attributed to the intestinal viscosity reduction, the substrate metabolism, and microbial cross-feeding mechanism (Choct, 1997; Bedford and Cowieson, 2012; Morgan et al., 2019). In the present study, the intestinal challenge was applied and higher nutrient digestibility, higher SCFA concentrations and better intestinal health, which presented better integrity and lower alterations, were observed when broilers were fed diets supplemented with a *B. subtilis* xylanase. The benefits observed in nutrient digestibility and intestinal health could explain the improvements on growth performance when the *B. subtilis* xylanase was added in the reduced energy diet. Further investigations are suggested to better understand the effects of this bacterial xylanase on the beneficial microorganism populations and its effects on intestinal health of broilers fed diets with different ingredients.

In conclusion, the supplementation of a *Bacillus subtilis* endo 1,4-β-xylanase in corn-soybean meal-based diets formulated with energy reductions improved nutrient digestibility and energy utilization, enhanced intestinal integrity and health and improved the growth performance in broilers chickens undergoing an intestinal challenge. Those results indicate that the bacterial xylanase offers a new strategy to improve feed efficiency and intestinal health, while compensating the effects of energy reduction of diets.

## Declaration of interests

The authors declare the following financial interests/personal relationships which may be considered as potential competing interests:

Catarina Stefanello reports financial support and article publishing charges were provided by Jefo Nutrition Inc. and Belfeed NV. Heitor V. Rios, Thais B Stefanello, Mariana L. Moraes, Marcia S. Vieira, Ludovic Lahaye report a relationship with Jefo Nutrition Inc that includes: employment. Raquel S. García and Christelle Boudry report a relationship with Belfeed N.V. that includes: employment The authors declare that they have no competing interests. The remaining authors declare that the research was conducted in the absence of any commercial or financial relationships that could be construed as a potential conflict of interest. If there are other authors, they declare that they have no known competing financial interests or personal relationships that could have appeared to influence the work reported in this paper.
